# REPS1 as a Potential Biomarker in Alzheimer’s Disease and Vascular Dementia

**DOI:** 10.3389/fnagi.2022.894824

**Published:** 2022-06-22

**Authors:** Jiefeng Luo, Liechun Chen, Xiaohua Huang, Jieqiong Xie, Chun Zou, Mika Pan, Jingjia Mo, Donghua Zou

**Affiliations:** ^1^Department of Neurology, The Second Affiliated Hospital of Guangxi Medical University, Nanning, China; ^2^Department of Neurology, The Affiliated Hospital of Youjiang Medical University for Nationalities, Baise, China; ^3^Department of General Medicine, The Second Affiliated Hospital of Guangxi Medical University, Nanning, China

**Keywords:** Alzheimer’s disease, vascular dementia, biomarker, REPS1, Ras signaling pathway

## Abstract

Vascular dementia (VD) and Alzheimer’s disease (AD) are common types of dementia for which no curative therapies are known. In this study, we identified hub genes associated with AD and VD in order to explore new potential therapeutic targets. Genes differentially expressed in VD and AD in all three datasets (GSE122063, GSE132903, and GSE5281) were identified and used to construct a protein–protein interaction network. We identified 10 modules containing 427 module genes in AD and VD. Module genes showing an area under the diagnostic curve > 0.60 for AD or VD were used to construct a least absolute shrinkage and selection operator model and were entered into a support vector machine-recursive feature elimination algorithm, which identified REPS1 as a hub gene in AD and VD. Furthermore, REPS1 was associated with activation of pyruvate metabolism and inhibition of Ras signaling pathway. Module genes, together with differentially expressed microRNAs from the dataset GSE46579, were used to construct a regulatory network. REPS1 was predicted to bind to the microRNA hsa_miR_5701. Single-sample gene set enrichment analysis was used to explore immune cell infiltration, which suggested a negative correlation between REPS1 expression and infiltration by plasmacytoid dendritic cells in AD and VD. In conclusion, our results suggest core pathways involved in both AD and VD, and they identify REPS1 as a potential biomarker of both diseases. This protein may aid in early diagnosis, monitoring of treatment response, and even efforts to prevent these debilitating disorders.

## Introduction

Age-related neurodegeneration affects more than 36 million people around the world. The most common cause of dementia is Alzheimer’s disease (AD), which shows insidious onset and leads to progressive deterioration of cognitive and physical function as well as mood. By 2050, more than 115 million around the world may be affected by AD (Reisberg et al., 1997). Various factors seem to affect risk of AD-related dementia, such as diabetes, hypertension, and smoking (Luchsinger et al., 2005). The pathogenesis of AD may even involve the individual’s own immune system: natural and adaptive immune responses involving monocytes, macrophages, neutrophils, and peripheral blood T cells may contribute to the disease (Polfliet et al., 2001; Ziegler-Heitbrock, 2007; Baik et al., 2014; Gate et al., 2020). Studies in Tg2576/p75 NTR ± mice suggest that reducing amyloid β accumulation can improve cognitive deficits (Jian et al., 2016), but no treatments are yet available to cure or prevent AD (Tan et al., 2014).

The second most common type of dementia is vascular dementia (VD; O’Brien and Thomas, 2015), which can coexist with AD and other age-related neurological disorders (Gorelick et al., 2011). As populations age, the overall incidence of dementia doubles every 5.1 years, that of AD doubles every 4.5 years, and that of VD doubles every 5.3 years (Jorm and Jolley, 1998). AD and VD appear to be closely related in terms of risk factors and symptoms (Ashraf et al., 2016). Better understanding of the etiology of both diseases, and exploration of the overlap between them, may help clinicians diagnose and treat them.

Such work can take the form of identifying appropriate biomarkers of the two diseases (Biomarkers Definitions Working Group, 2001), particularly molecules that can be assayed in a cost-effective, minimally invasive way, such as in blood. In AD, for example, levels of the microRNA (miRNA) miR-34a may aid in early diagnosis (Jian et al., 2017). The protein RBM8A may regulate many genes related to AD pathophysiology (Zou D. et al., 2019). Furthermore, we identified hub genes associated with molecular subtypes as potential biomarkers for AD, as well as candidate therapeutic targets (Ma et al., 2022). Identifying biomarkers for AD and VD may help individualize treatment for patients as a function of their precise symptoms.

In the present study, our aim was to search for biomarkers of AD and VD using bioinformatics and to explore their potential biological role in the two diseases.

## Materials and Methods

### Data Preprocessing

Gene expression data from the datasets GSE122063, GSE132903, GSE5281, and GSE46579 were downloaded from the Gene Expression Omnibus^1^ (Barrett et al., 2013). The data in GSE122063 were obtained using the GPL16699 platform and included 56 AD, 36 VD, and 44 healthy brain tissue samples. The age of patients at death (mean ± SD, year) were 78.6 ± 8.5 for healthy controls, 81.4 ± 10.1 for VD patients and 80.9 ± 7.4 for AD patients. These three groups in the GSE122063 dataset did not differ significantly in age, sex, or time to death. Data in GSE5281 were obtained using the GPL570 platform and included 87 AD and 74 normal brain tissue samples. The age of patients at death ranged from 63 to 102 years. Data in GSE132903 were obtained using the GPL10558 platform and included 97 AD and 98 non-dementia control samples. Age of patients at death range of 195 samples were 70–102 years. Data on miRNA expression in GSE46579 were obtained using the GPL11154 platform and included 48 AD patients and 22 normal blood samples.

**FIGURE 1:**
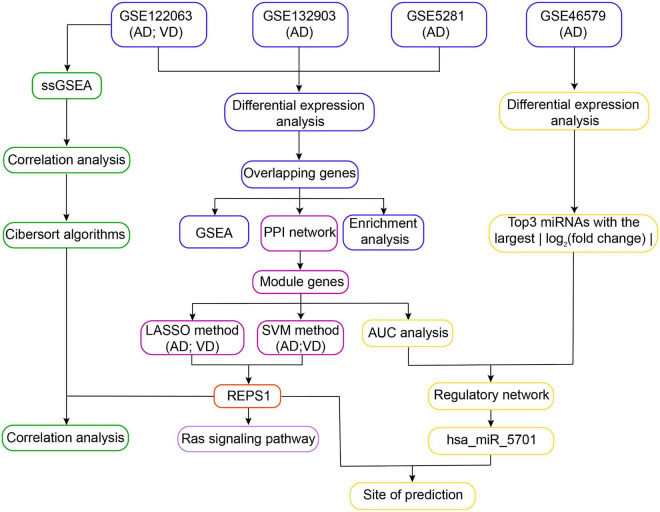
Workflow of the study. In *Step 1*, differentially expressed genes in Alzheimer’s disease (AD) and vascular dementia (VD) were identified in three datasets (GSE122063, GSE132903, and GSE5281). The differentially expressed genes overlapping across all three datasets were subjected to functional enrichment analysis and gene set enrichment analysis (GSEA). In *Step 2*, overlapping differentially expressed genes were used to construct a protein–protein interaction (PPI) network, leading to identification of module genes. REPS1 was identified as a hub gene using the least absolute shrinkage and selection operator (LASSO) and the support vector machine-recursive feature elimination (SVM-RFE) algorithm. In *Step 3*, module genes and the three differentially regulated microRNAs (miRNAs) showing the largest | log_2_ (fold change)| were used to construct a regulatory network. We found evidence that REPS1 binds hsa_miR_5701. In *Step 4*, single-sample gene set enrichment analysis was performed on the expression profiles of AD and VD in GSE122063. We also explored immune cell infiltration and correlation between REPS1 and biological pathways in AD and VD. AUC, area under the receiver operating characteristic curve.

Gene expression profiles were normalized using the “normalize Between Arrays” function of the limma package in R (Ritchie et al., 2015). The workflow for this study is shown in Figure 1.

### Analysis of Differential Gene Expression

Differentially expressed mRNAs (DEmRs) between AD and healthy individuals or between VD and healthy individuals were identified in datasets GSE122063, GSE5281, and GSE132903 using the limma package. DEmRs that were upregulated across all three datasets or downregulated across all three datasets, and whose differential expression was associated with an adjusted *P* < 0.05, were considered to be associated with dementia. The Himsc package in R was used to assess similarity of gene expression across different samples based on cumulative distribution curves.

### Functional Enrichment of Differentially Expressed Genes

We analyzed enrichment of DEmRs in Gene Ontology terms and Kyoto Encyclopedia of Genes and Genomes (KEGG) pathways using the clusterProfiler package in R (Yu et al., 2012). Enrichment was defined as *P* < 0.05. Interactions between DEmRs and cellular processes were explored using gene set enrichment analysis (GSEA; Mootha et al., 2003; Subramanian et al., 2005).

### Protein–Protein Interaction Network

A protein–protein interaction (PPI) network was constructed from intersecting genes whose k_core was greater than 500. The Molecular Complex Detection algorithm was performed to monitor PPI network modules with Cytoscape. From this network we identified module genes related to AD and VD.

### Feature Genes

The least absolute shrinkage and selection operator (LASSO) model and support vector machine-recursive feature elimination (SVM-RFE) algorithm were used to screen for feature genes. LASSO was performed within the glmnet package in R (Friedman et al., 2010). LASSO applies a penalty function to shrink the regression coefficient to 0 for variables that influence outcomes less. In this way, the method can converge toward a more accurate prediction model.

Support vector machine is a powerful binary classifier that establishes a classification hyperplane as a decision surface. SVM-RFE is an SVM-based machine learning method that reduces the eigenvectors generated by SVM in order to optimize the variables in a prediction model. The SVM-RFE algorithm was performed within the 1071 package in R (Huang et al., 2014), and potential biomarkers associated with *P* < 0.05 for diagnosing AD or VD were retained.

The ability of module genes to diagnose AD and VD was assessed by calculating the area under receiver operating characteristic curves (AUC) using the “coxph” function in the survival package (Barakat et al., 2019). Only module genes associated with AUC > 0.85 and *P* < 0.01 were retained.

### Network of MicroRNAs and Their Target Genes

We used the limma package to identify DEmiRs from the dataset GSE46579. For the three DEmiRs showing the largest | log (fold change)|, we predicted their potential target genes using TargetScan^2^. The resulting regulatory network was visualized using Cytoscape (Shannon et al., 2003), and potential functional aspects were explored using Metascape (Zhou et al., 2019).

### Single-Sample Gene Set Enrichment Analysis

Single-sample gene set enrichment analysis (ssGSEA) was used to explore the infiltration and activity of 24 immune cell types using marker gene sets (Bindea et al., 2013). The ssGSEA scores were standardized across immune cell types using the ‘‘normalizeBetweenArrays’’ function in the limma package. Radar plots were used to assess the potential correlations of immune cell infiltration between AD and VD. We used CIBERSORT^3^ to assess infiltration levels of immune cells. To identify genes associated with immune cell infiltration patterns, we calculated the immune cell types for the three datasets GSE122063, GSE132903, and GSE5281 using the limma package (Law et al., 2014).

### Statistical Analysis

The analyses of the present study were performed using the BioInforCloud platform. DEmRNAs and DEmiRs between AD and healthy individuals or between VD and healthy individuals were analyzed using an unpaired *t* test method within the limma package. Differences were considered significant if associated with *P* < 0.05.

## Results

### Identification of Differentially Expressed Genes in Vascular Dementia and Alzheimer’s Disease

**FIGURE 2:**
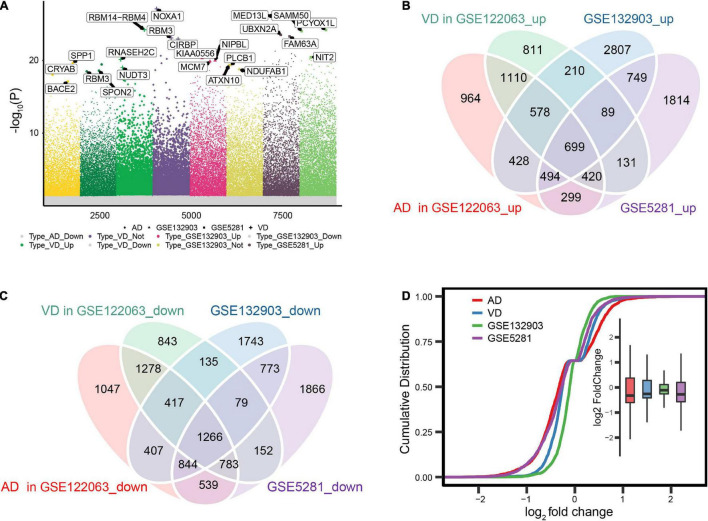
Differential gene expression in Alzheimer’s disease (AD) and vascular dementia (VD). **(A)** Differentially expressed genes were identified between controls and patients with AD or VD in the datasets GSE122063, GSE132903, and GSE5281. **(B, C)** Overlap of differentially expressed genes that were up- or downregulated in the two disorders. **(D)** Cumulative distribution plots showing similarity in the expression of differentially expressed genes across datasets.

We first identified differentially expressed genes among AD, VD, and control brain tissues (Figure 2A). A total of 1,965 differentially expressed genes overlapped across the three datasets (Figures 2B,C), of which 699 were consistently upregulated and 1,266 consistently downregulated in AD and VD. Expression levels of overlapping differentially expressed genes were most similar between AD and control samples in the datasets GSE122063 and GSE5281 (Figure 2D).

### Potential Functions of Overlapping Differentially Expressed Genes

**FIGURE 3:**
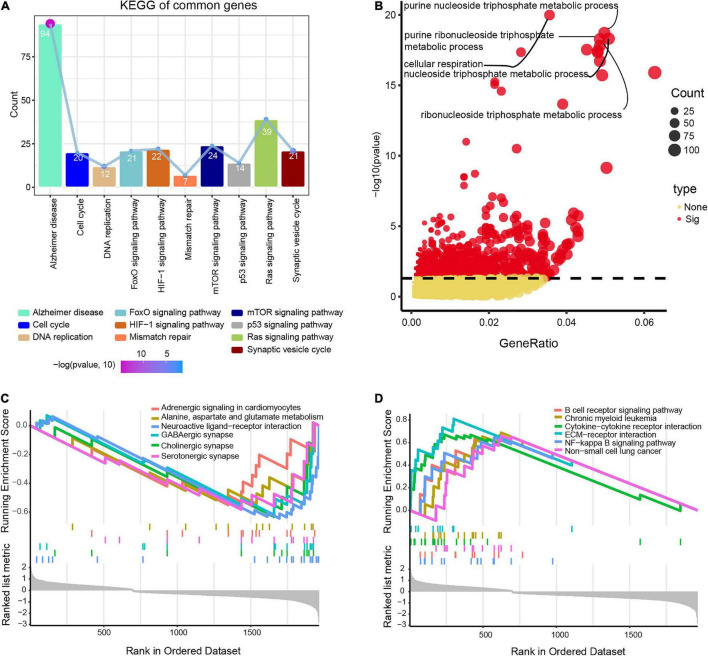
Potential functions of differentially expressed genes in Alzheimer’s disease (AD) and vascular dementia (VD). **(A)** Enrichment of genes in Kyoto Encyclopedia of Genes and Genomes (KEGG) pathways. **(B)** Enrichment of genes in Gene Ontology biological processes. **(C, D)** Lists of the top six pathways that the genes were predicted to **(C)** activate or **(D)** inhibit, based on gene set enrichment analysis. Enriched genes were defined as genes strongly expressed in AD/VD tissues that showed high enrichment scores.

Differentially expressed genes overlapping across the datasets GSE122063, GSE5281, and GSE132903 were enriched in biological processes involved in AD, mTOR signaling, and Ras signaling (Figure 3A), including purine nucleoside triphosphate metabolism, cellular respiration, and nucleoside triphosphate metabolism (Figure 3B).

Gene set enrichment analysis showed that the differentially expressed genes were involved in alanine aspartate and glutamate metabolism, cholinergic synapses and neuroactive ligand-receptor interactions, all of which were activated in AD (Figure 3C). Moreover, overlapping genes were predicted to inhibit B cell receptor signaling, interactions between cytokines and their receptors, and NF-kappa B signaling in AD (Figure 3D).

### Identification of Hub Genes Using Least Absolute Shrinkage and Selection Operator and Support Vector Machine-Recursive Feature Elimination

**FIGURE 4:**
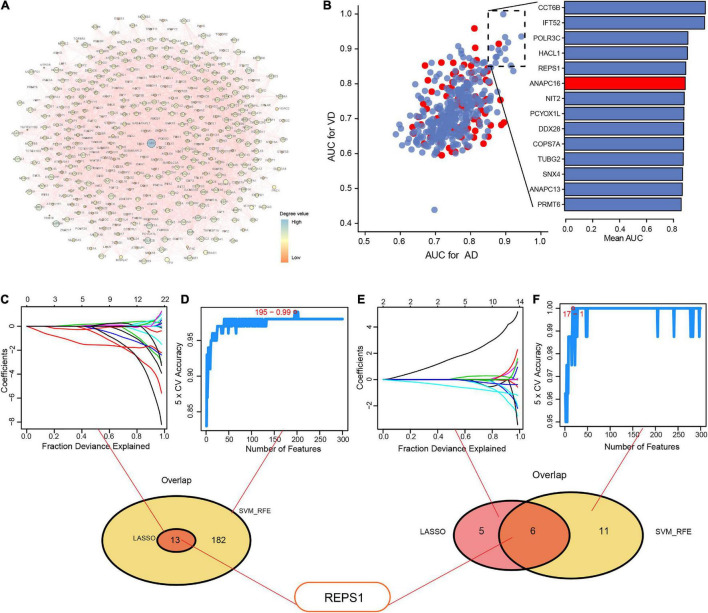
Identification of hub genes using LASSO and SVM-RFE. **(A)** Protein–protein interaction network to reveal module genes. **(B)** Evaluation of the diagnostic value of module genes involved in Alzheimer’s disease (AD) and vascular dementia (VD), based on the area under the receiver operating characteristic curve (AUC). Red indicates upregulated genes; blue, downregulated genes. **(C–F)** Module genes were entered into LASSO and SVM-RFE to obtain the overlapping feature genes in **(C, D)** AD or **(E, F)** VD. Feature genes are identified using different colors in **(C)** and **(E)**. LASSO, least absolute shrinkage and selection operator; SVM-RFE, support vector machine-recursive feature elimination.

The PPI network revealed 10 modules containing 427 module genes (Figure 4A), of which 14 showed AUC > 0.85 for both AD and VD (Figure 4B). From module genes in AD, LASSO identified 13 genes (Figure 4C) and SVM-RFE identified 182 genes (Figure 4D).

From the overlap between the genes identified by LASSO and SVM-RFE, we identified 13 feature genes: GLRX5, FDX1, TRMT11, GABBR1, SERPINA3, REPS1, IFIT2, ANAPC13, CASP7, DNAJC10, IFT52, TBL1Y, and NDUFV3.

From module genes in VD, LASSO identified 11 genes (Figure 4E) and SVM-RFE identified 17 genes (Figure 4F). From the overlap, we identified six feature genes: RAB5A, ALDOC, REPS1, KLHL21, PCF11, and CCT6B.

REPS1 appeared among the overlap genes base on both AD and VD, so we focused on it in subsequent bioinformatic analyses.

### Regulatory Network of DEmiRs in Alzheimer’s Disease

**FIGURE 5:**
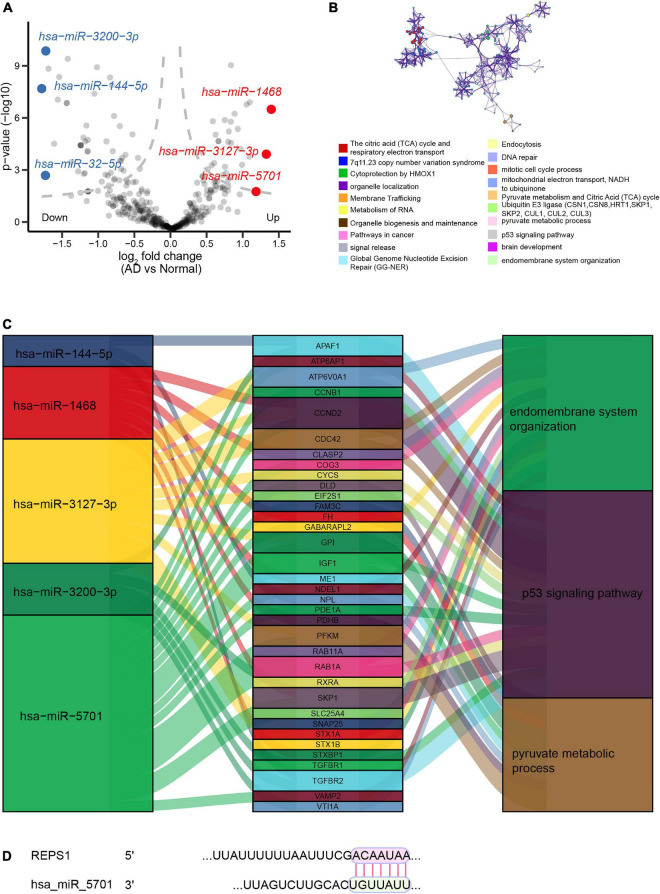
Regulatory network of miRNAs in Alzheimer’s disease (AD). **(A)** Volcano plot of differentially expressed miRNAs between AD and controls in the dataset GSE46579. Red indicates the top three upregulated miRNAs, while blue indicates the top three downregulated miRNAs. **(B)** Enrichment analysis of the three differentially expressed miRNAs showing the largest | log_2_ (fold change)| . **(C)** Sankey plots showing the biological functions potentially affected by the regulatory relationships between differentially expressed miRNAs and differentially expressed genes. **(D)** Predicted binding between REPS1 and hsa-miR-5701, based on TargetScan.

We identified 161 DEmiRs between AD and controls in the dataset GSE46579, of which 64 were upregulated and 97 were downregulated (Figure 5A). These DEmiRs were involved in RNA metabolism, organelle localization, the citric acid cycle and the respiratory electron transport (Figure 5B). The three DEmiRs showing the highest | log_2_ (fold change)| were involved in p53 signaling, pyruvate metabolism, and endomembrane system organization (Figure 5C). The DEmiR hsa-miR-5701 was predicted to bind to REPS1 (Figure 5D).

### Immune Cell Infiltration in Vascular Dementia and Alzheimer’s Disease

**FIGURE 6:**
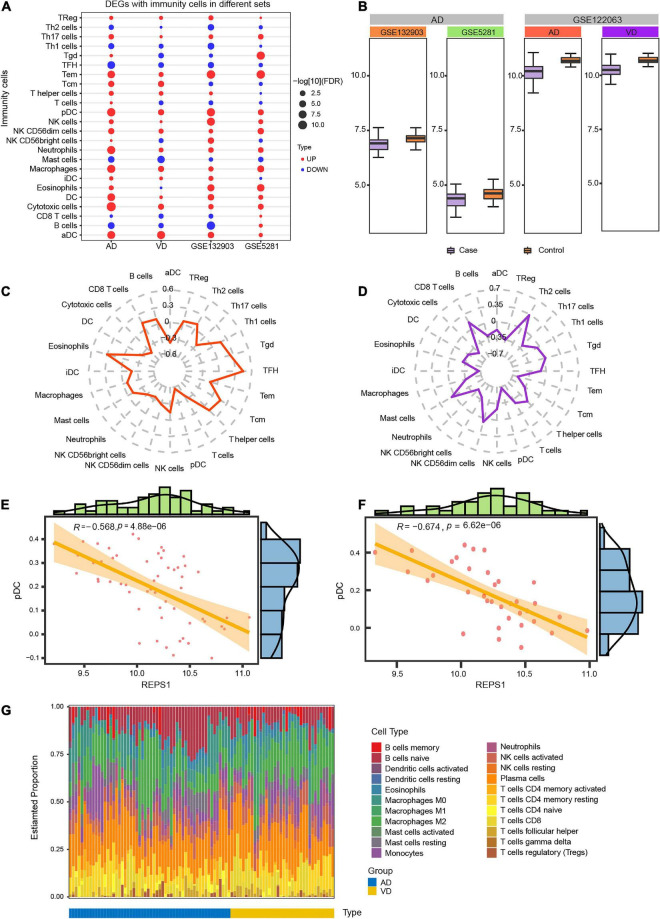
Degree of infiltration by immune cells in Alzheimer’s disease (AD) and vascular dementia (VD). **(A)** Differential infiltration by immune cells in the datasets GSE122063 (AD and VD), GSE132903, and GSE5281. **(B)** Expression of REPS1 in the datasets GSE122063 (AD and VD), GSE132903, and GSE5281. **(C, D)** Correlation of REPS1 expression with infiltration by 24 types of immune cells in AD and VD. **(E, F)** REPS1 negatively correlated with plasmacytoid dendritic cells (pDCs) in AD and VD. **(G)** Estimated proportions of 22 immune cell types in AD (blue) or VD (yellow).

Immune cell infiltration was compared between AD or VD patients and controls in the datasets GSE132903, GSE5281, and GSE122063. Patients showed significantly greater infiltration by Effective Memory T Cell (Tem), plasmacytoid dendritic cells (pDCs) and activated dendritic cells (aDCs; Figure 6A). REPS1, which was downregulated in patients relative to controls (Figure 6B), correlated positively with infiltration by follicular T helper cells and eosinophils in AD (Figure 6C), or with T helper 2 cells in VD (Figure 6D). Conversely, REPS1 correlated negatively with pDCs in AD (Figure 6E) and VD (Figure 6F). Plasma cells were the most abundant infiltrating cell type in AD and VD (Figure 6G and Supplementary Table 1).

### Potential Role of REPS1 in Vascular Dementia and Alzheimer’s Disease

**FIGURE 7:**
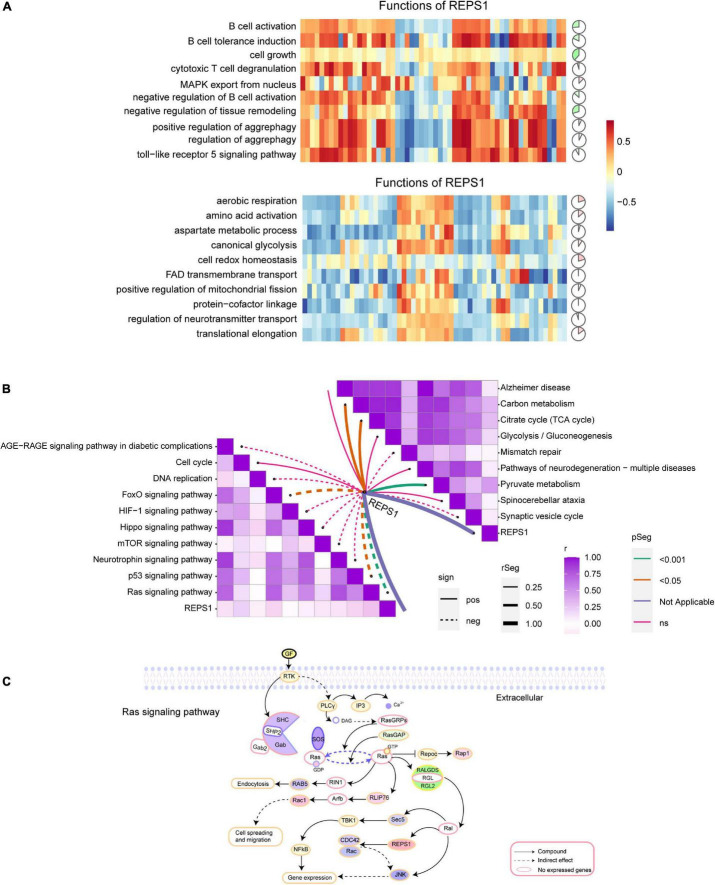
Correlation of REPS1 and pathways. **(A)** Correlations between REPS1 and biological processes. Red indicates activation, and green indicates inhibition. **(B)** Correlation between REPS1 and functional pathways. Solid lines indicate positive correlations, and dotted lines indicate negative correlations. **(C)** Schematic illustrating how REPS1 may trigger downstream Ras signaling.

In AD and VD, REPS1 correlated negatively with cell growth, and it was predicted to activate cellular redox homeostasis (Figure 7A). Based on KEGG pathway enrichment, REPS1 was predicted to activate pyruvate metabolism and to inhibit Ras signaling (Figures 7B,C).

## Discussion

AD and VD are age-related diseases that seriously affect quality of life. Age is a vital risk factor associated with AD (Armstrong, 2019), and as patients age, their disorder becomes more severe. In this study, REPS1 was identified as a potential biomarker of both disorders and Ras signaling as one of the drivers of pathogenesis. Given that neurodegeneration involves complex networks of interacting genes and pathways (Jian et al., 2021), we adopted a genomic rather than “candidate gene” approach to understanding the landscape of changes that may help explain AD and VD. Our results may aid in the early diagnosis, treatment and even prevention of these debilitating disorders.

Our analyses suggest that genes differentially expressed in AD and VD may be involved in NF-kappa B signaling and Ras signaling. These results are consistent with previous studies (Kirouac et al., 2017; Zou C. et al., 2019; Zhou et al., 2021). Furthermore, among that biological process further study is needed in order to determine which metabolic processes are up- or downregulated in AD or VD. LASSO or SVM-RFE methods identified 13 feature genes in AD samples. Among these, SERPINA3 (Norton et al., 2021), IFIT2 (Puig-Butille et al., 2014), and CASP7 (Zhang et al., 2019) have previously been proposed to play vital roles in AD. In contrast, we are unaware of previous studies linking AD to the genes REPS1, GLRX5, FDX1, TRMT11, GABBR1, ANAPC13, DNAJC10, IFT52, TBL1Y, or NDUFV3. Six feature genes overlapped between LASSO and SVM-RFE methods as associated with VD, and none of these genes has previously been linked to VD. These feature genes merit further study for their potential roles in AD or VD. As a key feature gene in AD and VD based on both LASSO and SVM-RFE methods, REPS1 became the focus of the present study. REPS1 is expressed ubiquitously; it is more abundant in heart and testis, and less abundant in brain, kidney, colon, and lung (Xu et al., 2001). Mutations in REPS1 can reduce palmitoylation as well as altered acylation in genetic diseases (Chamberlain and Shipston, 2015). Such alterations have been linked to AD and Huntington’s disease (Chavda et al., 2014). Moreover, REPS1 is predicted to be regulated by hsa_miR_5701, which emerged as one of the three DEmiRs showing the largest change in expression in AD and VD. This is the first report linking hsa_miR_5701 to neurological disease. It has already been linked to prognosis of patients with lung squamous cell carcinoma (Gan et al., 2019), and it can inhibit the proliferation of cervical cancer cells (Pulati et al., 2019).

Communication among different cell types in the central nervous system may contribute to AD (Vainchtein and Molofsky, 2020). We found evidence that aDCs and macrophages show substantial infiltration in both AD and VD. Dendritic cell-based therapy may be a more effective treatment for age-related diseases, such as AD (Cheng et al., 2015). The number of microglia, which act as phagocytic immune cells to clear material from the central nervous system, appears to decrease during AD progression (Hoozemans et al., 2011; Heneka, 2017). Furthermore, we observed here a negative correlation between pDCs and AD/VD. These considerations suggest that immune responses weaken over time in AD and VD. Furthermore, CIBERSORT analysis showed extensive infiltration by plasma cells in AD/VD. Age-dependent plasma cell accumulation may contribute to cognitive decline and to behavioral or neurodegenerative disorders in humans (Hepworth et al., 2021).

The function of REPS1 appears to depend on its Eps15 homology domain, which mediates PPIs (Nakashima et al., 1999). REPS1 may interact *via* this domain with downstream effectors, such as the Ras-Ral signaling pathway (Datson et al., 2011). We found REPS1 to correlate positively with pyruvate metabolism and the citrate cycle, both of which have been linked to neuroinflammation and neurodegeneration (Garabadu et al., 2019). We also found REPS1 to correlate positively with the cell cycle. Dysregulation of the cell cycle is a cancer hallmark, and early disruptions in the cell cycle in AD may cause oncogenic signal transduction (Raina et al., 2000). Conversely, we found that REPS1 correlated negatively with Ras signaling. PKC–Ras signaling can be upregulated to compensate for loss of M1 muscarinic acetylcholine receptor activity and thereby mitigate dementia symptoms in elderly (Chen et al., 2020), and our results imply that the same may be true for AD. REPS1 interacts with Ral protein, proteins that activate Cdc42 and Rac GTPases that inhibit JNK, so our results implicate all these proteins in AD and VD.

Our findings should be interpreted with caution because they are based entirely on bioinformatic analyses. Experimental studies should explore whether levels of REPS1 protein change in the brains of individuals with VD or AD.

## Conclusion

In this study, we identified REPS1 as a potential biomarker of AD and VD and as a candidate therapeutic target. Our results also implicate Ras signaling in the two neurodegenerative disorders.

## Data Availability Statement

The original contributions presented in this study are included in the article/Supplementary Material; further inquiries can be directed to the corresponding authors.

## Ethics Statement

Ethical review and approval was not required for the study on human participants in accordance with the local legislation and institutional requirements. Written informed consent for participation was not required for this study in accordance with the national legislation and the institutional requirements. Written informed consent was obtained from the individual(s) for the publication of any potentially identifiable images or data included in this article.

## Author Contributions

DZ and JM conceived and designed the study. JL, LC, and XH performed analyses as well as collected and analyzed the data. All authors prepared the figures and tables, and wrote the manuscript. All authors reviewed the manuscript and approved its submission.

## Conflict of Interest

The authors declare that the research was conducted in the absence of any commercial or financial relationships that could be construed as a potential conflict of interest.

## Publisher’s Note

All claims expressed in this article are solely those of the authors and do not necessarily represent those of their affiliated organizations, or those of the publisher, the editors and the reviewers. Any product that may be evaluated in this article, or claim that may be made by its manufacturer, is not guaranteed or endorsed by the publisher.

## Footnotes

1
https://www.ncbi.nlm.nih.gov/geo/

2
www.targetscan.org/vert_72

3
https://cibersort.stanford.edu/

## Supplementary Material

Supplementary Table 1 Estimated proportions of 22 immune cell types in AD.
